# A Case of a Negative Urine Pregnancy Test in a Multiple Gestation Pregnancy

**DOI:** 10.7759/cureus.30725

**Published:** 2022-10-26

**Authors:** Joshua Reitz, Brittany C Hartman, Michael E Chase, Dylan Krause, Alexis L Cates

**Affiliations:** 1 Emergency Medicine, Einstein Medical Center Philadelphia, Philadelphia, USA; 2 Emergency Medicine, Ochsner Medical Center, New Orleans, USA

**Keywords:** urine pregnancy test, prozone phenomenon, β-hcg, hook effect, multiple gestation, false negative urine pregnancy test

## Abstract

Urine pregnancy tests (UPTs) are a highly reliable method of detecting pregnancy, with reported 100% sensitivity and 99.2% specificity. This test relies on the detection of β-human chorionic gonadotropin (β-hCG) molecules in the urine through a two-site sandwich immunoassay. Although a nearly perfect test, it is common knowledge that this test can be falsely negative if performed too early in the pregnancy when urinary β-hCG concentrations fall below detectable levels. Less commonly known is that the test may provide a false-negative result when urinary β-hCG concentrations are extremely elevated, such as gestational trophoblastic disease or multiple gestations. Here, we present a case of a patient with a prior positive urine pregnancy test who presents with symptoms consistent with early pregnancy. Repeat testing resulted in a negative urine pregnancy test. Additional workup revealed significantly elevated serum quantitative β-hCG and bedside ultrasound revealed multiple gestation intrauterine pregnancy. The patient ultimately delivered triplets by repeated caesarean section. It is important for physicians to understand and recognize the limitations of the urine pregnancy test in order to best facilitate care for patients who may have a false-negative pregnancy test result, as there are significant risks of improper patient management with a multiple gestation pregnancy or gestational trophoblastic disease.

## Introduction

Urine pregnancy tests (UPTs) are used to detect the presence of human chorionic gonadotropin (hCG) in the urine and, when positive, are highly suggestive of pregnancy. UPTs are widely available, reliable, relatively inexpensive, and offer quick results. Understanding the mechanism by which UPTs produce their results is important to understanding the accuracy and reliability of this testing modality. Occasionally, UPTs produce false results, which can alter the management of the patient in question and may lead to delays in diagnosis.

Human chorionic gonadotropin hormone

Human chorionic gonadotropin (hCG) is produced almost exclusively by the trophoblast, more specifically by the cytotrophoblast and syncytiotrophoblast, of the placenta once implantation has occurred. hCG is a glycoprotein composed of α- and β-subunits [1]. The α-subunit of hCG is identical to the α-subunit of other hormones in the glycoprotein family, including luteinizing hormone (LH), follicle-stimulating hormone (FSH), and thyroid-stimulating hormone (TSH) [2]. These hormones could potentially have cross-reactivity with the α-subunit of hCG. Therefore, UPTs specifically detect the β-subunit of hCG, or β-hCG [3].

To further complicate matters, the β-subunit exists in several different forms. These include hyperglycosylated hCG (H-hCG), nicked hCG, free β-subunit, the core fragment of β-hCG (hCG-βcf), and others. The relative concentrations of each different form change throughout the course of pregnancy. For example, H-hCG is the predominant form of β-hCG produced after implantation but accounts for less than 5% of β-hCG in the second and third trimesters. On the other hand, hCG-βcf is highest in mid-pregnancy urine [4].

How do UPTs work?

UPTs are a qualitative, two-site sandwich immunoassay [3] that works by binding β-hCG molecules in the urine [1]. The positive threshold is typically between 5 and 50 mIU/mL, but this can vary based on the specific immunoassay in use. A positive test is characterized by a color change [5].

The immunoassay for the UPT is based on the lateral flow principle. In this type of test, urine is placed into a sample introduction window. If β-hCG is present in the urine, it binds to a complementary anti-hCG antibody attached to a gold particle. The hCG-antibody-gold conjugate will move down the test strip to meet a second antibody that has been fixed to the test strip, which is complementary to β-hCG, resulting in a color change at the test line. Antibody-gold conjugates that do not bind to hCG continue to flow down the strip and bind at the control line, also causing a color change. A positive urine pregnancy test occurs when both the test and control lines are observed. A negative result occurs when the control line is observed alone [6].

Urine β-hCG tests have been reported to achieve as high as 100% sensitivity and 99.2% specificity in a clinical setting, as well as a positive predictive value of 98.3% and a negative predictive value of nearly 100% [2].

The most common cause of a false-negative UPT is performing the test too soon after conception [1]. Additional etiologies of false-negative UPTs include, but may not be limited to, multiple gestation, molar pregnancies, and trophoblastic disease. In cases of multiple gestations and gestational trophoblastic disease, the etiology of false-negative UPTs is suspected to be due to very high urine hCG concentrations, resulting in the hook effect or prozone phenomenon [6].

## Case presentation

A 34-year-old black female with no chronic medical conditions initially presented to an outside emergency department (ED) reporting several days of nausea and vomiting. She was actively trying to conceive without the use of reproductive technology, and her last menstrual period (LMP) was approximately eight weeks prior. She had three prior uncomplicated pregnancies and live births. Testing at the outside ED was remarkable for a positive UPT. She was treated and diagnosed with nausea and vomiting during pregnancy, with an estimated gestational age (EGA) of eight weeks by the date of LMP. The patient was provided a prescription for antiemetics and instructed to follow up with obstetrics to establish care and obtain a confirmatory ultrasound.

Several weeks later, the patient presented to a second ED where she reported three weeks of nausea and vomiting which had acutely worsened over the previous several days despite the use of prescribed antiemetics. She had not yet established care with obstetrics or obtained a confirmatory ultrasound. She denied abdominal discomfort, pelvic cramping, or vaginal bleeding since her initial ED evaluation. Initial workup revealed a negative UPT. When informed, the patient provided a copy of the discharge paperwork from the previous ED, which confirmed a previously positive UPT. Given the information provided, it was suspected that the UPT was falsely negative. Bedside transabdominal ultrasound was performed and revealed at least two gestational sacs and two fetal poles, thought to be a twin gestation. Fetal heart rates were reassuring, and measurements revealed EGA between nine and ten weeks for both. The patient was informed of the suspected multiple gestations. The lab workup included quantitative serum β-hCG, which resulted in 347,737 mIU/mL. Although quantitative serum β-hCG was markedly elevated, given the patient's EGA and visualized multiple gestations, there was low suspicion for concurrent molar pregnancy or gestational trophoblastic disease. Additionally, given her suspected EGA of 10 weeks, her quantitative serum β-hCG levels were within normal limits for a multiple gestation pregnancy. Additional lab workups including complete blood count, basic metabolic panel, and urinalysis revealed no abnormalities. The patient's nausea had resolved after treatment with intravenous (IV) fluid rehydration and IV antiemetics. She was subsequently able to tolerate oral intake while in the ED. Maternal-fetal medicine was contacted while the patient was in the ED and facilitated scheduling a follow-up appointment. She was prescribed a different antiemetic and was provided appropriate return precautions and outpatient maternal-fetal medicine follow-up instructions.

At the outpatient confirmatory ultrasound, the patient was found to have a dichorionic-triamniotic triplet gestation. After an otherwise uncomplicated pregnancy, the patient was successfully delivered by repeated cesarean section at 34 weeks and five days. Delivery confirmed a dichorionic triamniotic pregnancy. Baby A was single-amnion, single-chorion, weighed 1.92 kg and had APGAR scores of 7 and 9. Baby B was a single amnion, shared chorion, weighed 1.94 kg, and had APGAR scores of 3, 6, and 7. Baby C was born with a single amnion, shared chorion, weighed 1.84 kg, and had APGAR scores of 9 and 9.

## Discussion

**Figure 1:**
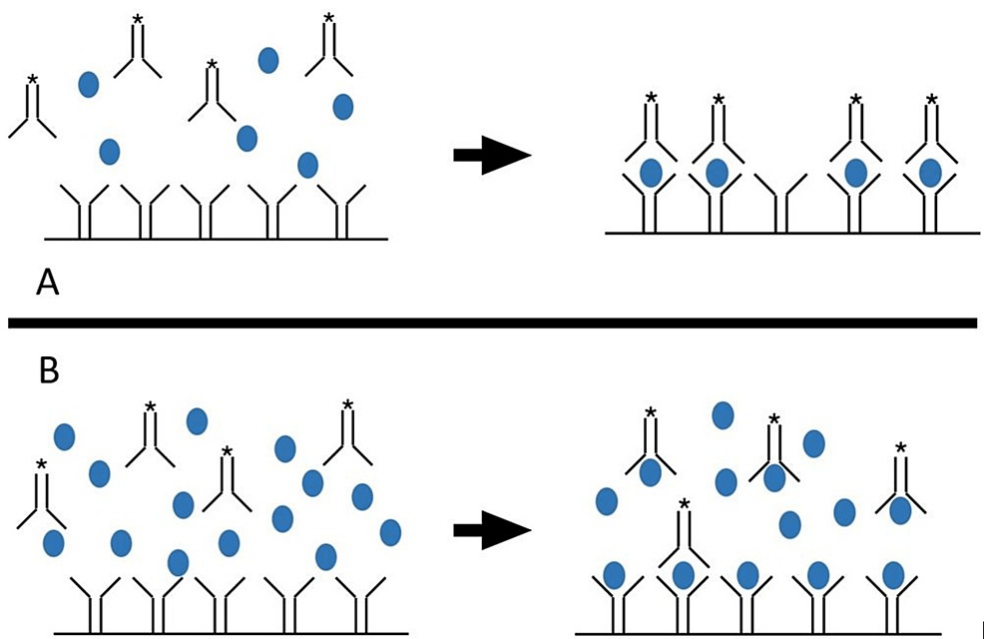
Urine pregnancy test mechanism A: Ab-hCG-Ab* sandwich formation with normal hCG concentration, results in positive UPT; B: high concentration of hCG resulting in inability to form Ab-hCG-Ab* sandwich, results in falsely negative UPT.

The hook effect, also known as high-dose hooking or the prozone phenomenon, occurs in immunometric assays when β-hCG concentrations are so high that the formation of the Ab-hCG-Ab* sandwich cannot take place (Figure 1) [1]. This typically occurs when the β-hCG concentrations are greater than 5,000 to 20,000 mIU/mL. At high concentrations of β-hCG, both fixed and free-floating antibodies are bound to β-hCG, preventing the sandwich formation and resulting in a negative test [4].

**Figure 2:**
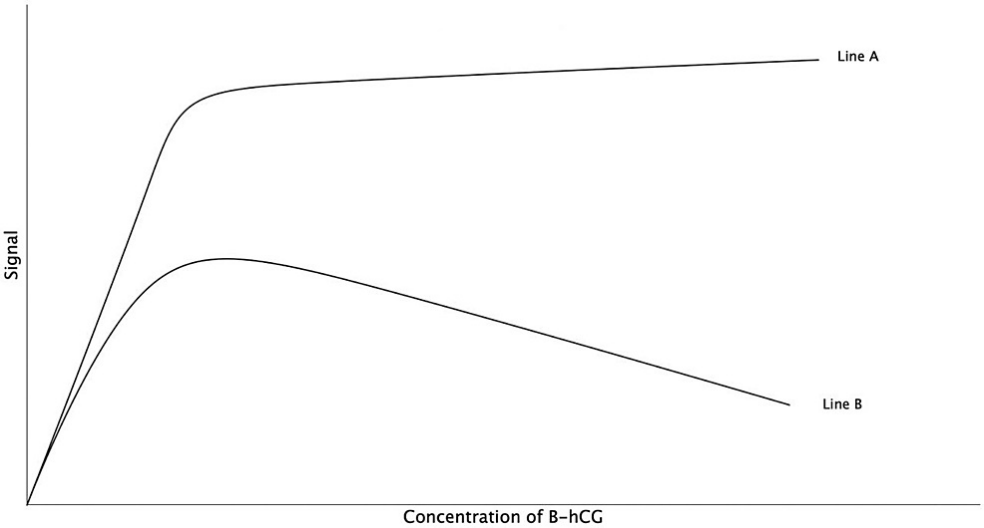
The hook effect Line A represents the expected graph, where an increase in concentration is associated with an increase in signal. Line B represents the hook effect, where an increase in concentration is associated with a decrease in the signal [4].

The hook effect produces a false-negative test result despite having high concentrations of β-hCG. When the concentration of β-hCG is plotted against a positive test result on a graph, the shape is "hook-like," hence the name hook effect (Figure 2). The expected result of increasing β-hCG concentration would be an increase in signal (line A), however, with the hook effect, the opposite occurs and there is a decrease in signal with an increase in concentration (line B).

There is also a "hook-like" effect, which has been characterized in the literature. This hypothesis is based on the fact that other variants of hCG are present in the urine sample. One, in particular, hCG-bcf, has been known to be present in high concentrations at certain stages of pregnancy. When this occurs, it saturates the free antibodies and prevents sandwich formation [4]. This phenomenon was recently confirmed when previously unavailable purified hCG-bcf was added to a pregnant patient’s urine and resulted in a negative test [6].

Detection of hCG is useful in the evaluation of pregnancy, molar pregnancy, and trophoblastic disease. All of these entities produce hCG at varying levels, which is reportedly detectable on UPTs [7]. Total levels of hCG greater than 100,000 mIU/mL in early pregnancy are highly suggestive of a complete hydatidiform mole, although many normal pregnancies may reach this level around 8-11 weeks of gestation [7].

Though a rare phenomenon, it is important for physicians to be aware that the hook effect has been reported to occur in molar pregnancies, trophoblastic disease, and multiple gestation pregnancies. False-negative results associated with these diagnoses could potentially lead to delayed diagnosis, improper follow-up, and improper patient management. A negative UPT result in a patient with a suspected pregnancy should be further evaluated with a quantitative serum β-hCG and/or an ultrasound. It is important for emergency medicine physicians, who frequently see patients early in pregnancy, to be aware of this phenomenon as a limitation of the urine pregnancy test and recognize that inaccurate results might affect patient management.

## Conclusions

Urine pregnancy tests are nearly perfect, but they can produce false-negative results when urinary β-hCG concentrations are significantly increased or decreased. One suggested reason for the false-negative urine pregnancy test in significantly elevated β-hCG levels, such as in cases of multiple gestations, is known as the hook effect. Physicians should be aware of this phenomenon as failure to recognize false-negative results has the potential to result in inappropriate patient management and subsequent negative patient outcomes.
